# Unique Fine Morphology of Mouthparts in *Haematoloecha nigrorufa* (Stål) (Hemiptera: Reduviidae) Adapted to Millipede Feeding

**DOI:** 10.3390/insects11060386

**Published:** 2020-06-22

**Authors:** Yan Wang, Junru Zhang, Wanshan Wang, Jolanta Brożek, Wu Dai

**Affiliations:** 1 Key Laboratory of Plant Protection Resources and Pest Integrated Management of the Ministry of Education, College of Plant Protection, Northwest A&F University, Yangling 712100, Shaanxi, China; wangyan105422@163.com (Y.W.); millipedeassassin@gmail.com (J.Z.); wangwanshang1004@163.com (W.W.); 2 Faculty of Natural Science, Institute of Biology, Biotechnology and Environmental Protection, University of Silesia in Katowice, Bankowa 9, 40-007 Katowice, Poland; jolanta.brozek@us.edu.pl

**Keywords:** Reduviidae, mouthparts, sensillum, feeding, predation

## Abstract

Millipede assassin bugs are a diverse group of specialized millipede predators. However, the feeding behavior of Ectrichodiinae remains poorly known, especially how the mouthpart structures relate to various functions in feeding. In this study, fine morphology of the mouthparts and feeding performance of *Haematoloecha nigrorufa* (Stål, 1867) was observed and described in detail for the first time. The triangular labrum is divided by a conspicuous transverse membrane into a strongly sclerotized basilabrum and a less sclerotized distilabrum. Fifteen types of sensilla are distributed on the mouthparts. Each mandibular stylet has an expanded spatulate apex and about 150 approximately transverse ridges on the external middle side; these help in penetrating the ventral trunk area and the intersegmental membranes of millipede prey. The right maxilla is tapered. On the internal surface are a row dorsal short bristles near the apex and a row of ventral bristles preapically. A longitudinal row of long lamellate structures extend proximate for a considerable distance, lie entirely within the food canal, and bear several short spines and short bristles. There is no obvious difference between males and females in the distribution, number, and types of sensilla on mouthparts. The adult feeding process involves several steps, including searching and capturing prey, paralyzing prey, a resting phase, and a feeding phase. The evolution of the mouthpart morphology and the putative functional significance of their sensilla are discussed, providing insight into the structure and function of the mouthparts adapted for millipede feeding.

## 1. Introduction

Mouthparts are the feeding organs of insects [1,2,3] and morphological variation in mouthparts generally corresponds well to their different feeding requirements [4,5]. A large number and variety of sensilla are attached to the different mouthparts, which play important roles in host search, detection, feeding, and mating [6,7,8,9]. The types and quantities of various sensilla are closely related to the feeding habits of insects. The mouthpart complex in Hemiptera is often called the “rostrum”, “sucking beak”, or piercing-sucking mouthparts [10]. During the long-term evolutionary history of Hemiptera, the components of this feeding apparatus have been clearly modified to serve their unique functions in different groups of bugs [11,12,13,14,15,16] and specialized to various sources of food [17]. Heteroptera, as compared to other groups of hemipterans (Cicadomorpha, Fulgoromorpha, Sternorrhyncha, and Colleorrhyncha) display a wider array of trophic and morphological diversity [12].

The sensillar systems of heteropteran mouthparts have been studied in representatives across different trophic groups and different families often display specific characteristics under examination [15,16,18,19,20,21,22,23,24,25]. Although data on some aspects of mouthpart morphology of Heteroptera are abundant, based on light and scanning electron microscopy, previous observations have been reported at various levels of accuracy [12,26,27,28,29,30,31,32,33,34,35,36]. Thus, consistent and detailed studies are needed in order to provide useful comparative data.

The feeding behavior of Hemipterans has been inferred from mouthpart structures [31,32], or was based on electropenetrograph (EPG) apparatus [37,38]. Unfortunately, ethological details about feeding behavior are lacking [39,40,41]. The fine morphology of mouthparts allows for us to interpret the function of the component parts of the feeding apparatus and provides us with information to understand the actual feeding mechanism.

Assassin bugs, or Reduviidae (Insecta: Heteroptera), are the second largest family and one of the most morphologically diverse groups of true bugs, with more than 6600 species [42]. Some data suggest that the evolutionary transition to predation in this family coincided with the reduction of one segment of their labium [43]. Generally, the reduviid rostrum is three-segmented, short and thick, usually curved and arched, and, when at rest, the labium is held in a longitudinal groove (the friction groove) in the center of the anterior thoracic and abdominal plates.

Details of mouthpart morphology of reduviid species have received only sporadic. Previous studies have mostly concentrated on the terminal labial sensilla [12,18,44], gross morphology of the mandible and maxilla [45,46], interlocking mechanisms of maxillae and mandibles [47], and gross morphology of the labium and labrum [46,48,49,50,51,52]. More detailed information on fine structure is needed to determine how much variability in mouthparts occurs across different taxa, particularly those with relatively narrow feeding specialization.

Millipede assassin bugs (Hemiptera: Reduviidae: Ectrichodiinae) are a diverse group with more than 700 species known worldwide [52]. Many ectrichodiines appear to be specialized millipede (Diplopoda) predators, but details of the predator-prey relationships, including prey specificity and point of mouthpart insertion are largely undocumented [53]. *Haematoloecha nigrorufa* (Stål, 1867) is a common and widespread species of assassin bug in China (Beijing, Zhejiang, Sichuan, Fujian, Jiangxi, Jiangsu, Gansu, Liaoning, Shaanxi) and some other Asian countries (Japan, Korean Peninsula), mainly feeding on millipedes and some arthropod pests. The fine structure of the mouthparts of *H. nigrorufa*, and the significance of these mouthpart structures for feeding, have not been previously studied.

This paper describes morphological observations of the mouthparts and feeding performance of *H. nigrorufa* to expand our knowledge of modifications of these organs and their sensilla system in a species that is adapted to the difficulties of obtaining food from millipedes. We focused on sensilla typology, distribution, and possible functions with the aim of identifying more character sets that are useful for future comparative morphological studies in Reduviidae.

## 2. Material and Methods

### 2.1. Insect Collecting

Adults of *H. nigrorufa* used for SEM in this study were collected with sweep nets at the campus of Northwest A&F University in Yangling, Shaanxi Province, China (34°160 *n*, 108°070 E, elev. 563 m) in August 2019, preserved in 75% ethanol, and stored at 4°C. For observing the performance of the mouthparts during feeding inside different types of prey, additional adults of *H. nigrorufa* were collected at the same locality in September 2019. Prey animals were not dissected after feeding.

### 2.2. Samples for SEM

Adult males (*n* = 4) and females (*n* = 12) were dipped into 10% NaOH solution for 2h and cleaned twice while using an ultrasonic cleaner (KQ118, Kunshan, China) 15s each time. Dehydration used serial baths of 80%, 90% and 100% ethanol each for 15 min. The materials were air dried, coated with a film of gold (Q150T-S, Quorum, West Sussex, UK), and then imaged with a Nova Nano SEM-450 (FEI, Hillsboro, OR, USA) at 5–10 kV in the scanning microscopy laboratories of the Life Science Research Core Services of Northwest A & F University.

### 2.3. Feeding Behavior on Millipedes

The observation of predatory behavior in *H. nigrorufa* lasted for two months and insects were bred in a transparent plastic basin 150 mm diameter, 50 mm tall. The bottom of the basin was covered with cotton wool 15 mm high, which was moistened with about 50 ml of water. On the cotton wool, bark and some leaves were placed to simulate the natural environment. The feeding conditions were (24 ± 1) ℃, RH (70 ± 15) %. Mealworms, armyworms, earwigs, polydesmid millipedes, juliform millipedes, and spirostreptid millipedes were separately used as prey. After 72 hours of fasting, the prey was offered to 20 adult individuals of *H. nigrorufa* in the basin and observations of the results. The predatory behavior was captured by a Nikon D500 camera and the images were imported into a computer for later analysis.

### 2.4. Image Processing and Morphometric Measurement

Photographs and SEMs were observed and measured after being imported into Adobe Photoshop CC 2019 (Adobe Systems, San Jose, CA, USA).

### 2.5. Data Analysis

The lengths of the mouthpart were compared between sexes using Student t-test. Statistical analyses were executed using SPSS 19.0 (SPSS, Chicago, IL, USA).

### 2.6. Terminology

The sensilla were classified according to their external morphology, length, distribution, and position. The terminology of sensilla follows Altner and Prillinger [54] and Frazier [55], with less specialized nomenclature from Brożek and Chłond [18]. Terminology of the labium and stylet bundle structures follow Weirauch [42,43], Cobben [12], Brożek, and Herczek [47], with some new terms established based on the present study.

## 3. Results

### 3.1. General Morphology of Mouthparts

**Figure 1:**
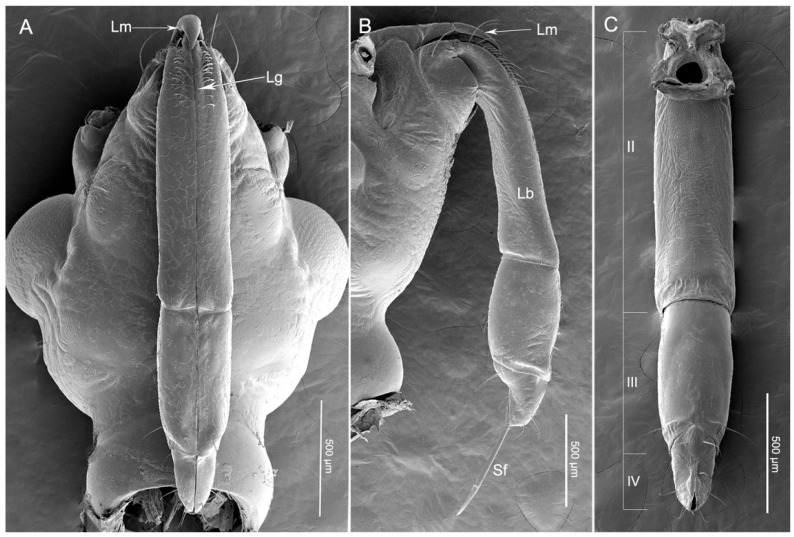
Scanning electron micrographs of the head of *Haematoloecha nigrorufa*. (**A**). Ventral view; (**B**). Lateral view; (**C**). Dorsal view showing three-segmented labium (II–IV); Sf, stylet fascicle; Lm, labrum; Lb, labium; Lg, labial groove.

The main elements of the mouthparts of *H. nigrorufa* are similar to other reduviid species and include the two-part labrum, three-segmented labium (the order of segment numbers in this group is from II–IV) (Figure 1A–C), and stylet fascicle (Sf) composed by two separated mandibular stylets (Md) and two interlocked maxillary stylets (Mx) (Figure 1B). No obvious differences were noted between the mouthpart structure of females and males except for their length (t(13) = 2.235, *p* = 0.044) (Table 1).

### 3.2. Labrum

**Figure 2:**
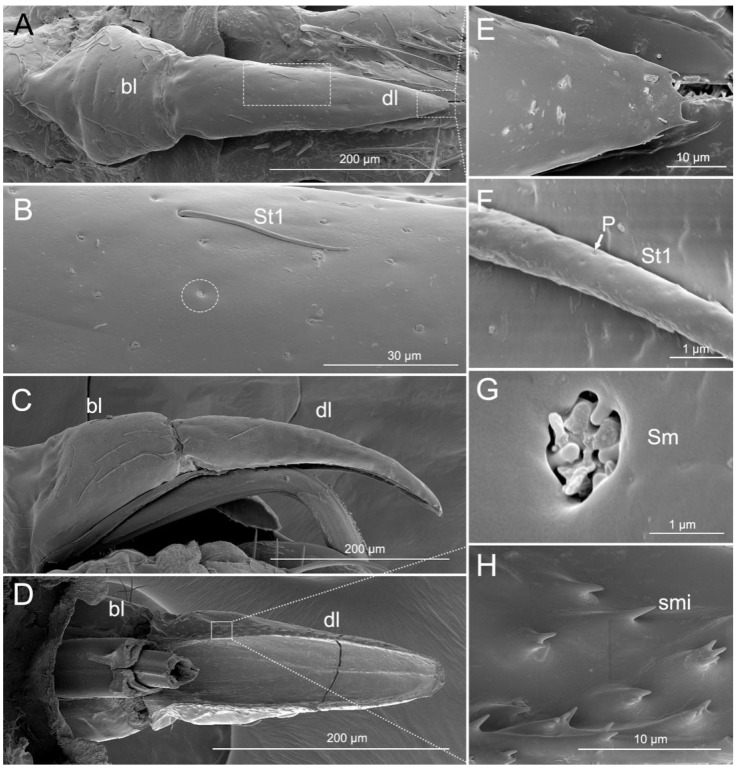
SEM of labrum of *Haematoloecha nigrorufa*. (**A**). Ventral view; (**B**). Enlarged view of box in (**A**), showing sensilla trichodea (St1) and multilobular sensilla (Sm); (**C**). Lateral view; (**D**). Dorsal view; (**E**). Enlarged view of tip of labrum; (**F**). Enlarged view of surface of sensilla trichodea (St1) showing pores (*p*); (**G**). Enlarged view of multilobular sensilla (Sm); (**H**). Enlarged view of box in (**D**), showing spike-like microtrichia (smi); bl, basilabrum; dl, distilabrum.

The triangular labrum reaches the base of the second labial segment (Figure 1A,B and Figure 2A). The labrum is divided by a conspicuous transverse membranous zone into two unequally sclerotized parts: a strongly sclerotized basilabrum (bl) and a less sclerotized distilabrum (dl) (Figure 2A,C,D). The basilabrum is wide and it accounts for 1/3 of the length of the labrum. The surface of the basilabrum is slightly plicated and sensilla trichodea (St1) and multilobular sensilla (Sm) are sparsely distributed on it (Figure 2A,C). The distilabrum (dl) is elongated and cone-shaped. In this part of the labrum, sensilla trichodea (St1) and multilobular sensilla (Sm) are less numerous. Moreover, several small spikes (smi) were observed at the tip of the distilabrum (dl) (Figure 2E,H). On the ventral side of the distilabrum there is a groove (gr) that holds a proximal part of the stylet fascicle (Sf) (Figure 2D). Spike-like microtrichia (smi) are irregularly distributed on the ventral surface (Figure 2D,H). Sensilla trichodea I (St1) are at mid-length, slightly curved, and lay flat on the surface of the labrum (Table 2, Figure 2B). The base of the sensilla is set in a pit, the surface has many pores and the tip is rounded (Figure 2B,F). Multilobular sensilla (Sm) are very small, but numerous, placed in cuticular cavities and resemble spread fingers (Table 2, Figure 2B,G).

### 3.3. Labium

The assassin bug *H. nigrorufa* is similar to most other reduviids in having a labium with only three segments. The short and stout three-segmented (II–IV) labium encloses the two mandibular stylets (Md) and two maxillary stylets (Mx) in the labial groove (Lg) (Figure 1B). The groove (Lg) is a shallow depression that is situated on the anterior side of segments two to four. The edges of the groove are close together and form one straight suture above the stylets (Figure 1A). The labial segments differ in morphology and size (Table 1, Figure 1A–C).

**Figure 3:**
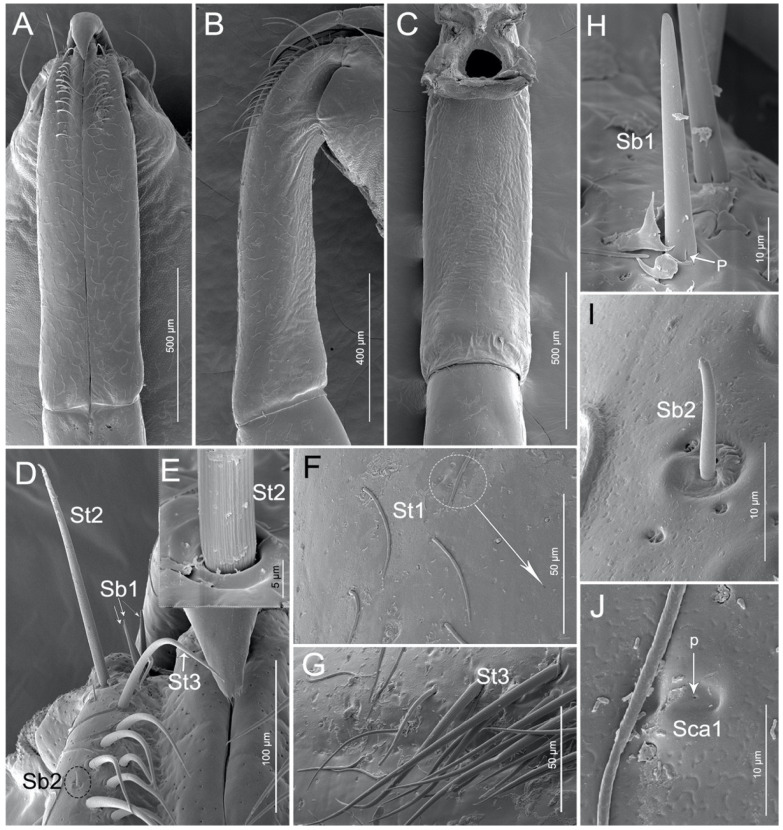
SEM of second labial segment of *Haematoloecha nigrorufa*. (**A**). Ventral view; (**B**). Lateral view; (**C**). Dorsal view; (**D**). Base of the second segment showing sensilla trichodea (St2), sensilla trichodea (St3), sensilla basiconica (Sb1) and sensilla basiconica (Sb2); (**E**). Base view of sensillum trichodeum (St2); (**F**). Enlarged view of dorsal surface of labium showing sensilla campaniformia (Sca1) and sensilla trichodea (St1); (**G**). Sensilla trichodea (St3); (**H**). Sensilla basiconica (Sb1); (**I**). Sensilla basiconica (Sb2); (**J**). Sensilla campaniformia (Sca1); *p*, pore.

Following previous authors we interpret the first labial segment (I) to be either lost or fused to the head capsule. The first visible segment is the second segment (II) and it is the longest segment of the labium (Table 2, Figure 1A–C). The proximal part is slightly narrowed and abundant sensilla are concentrated on this area (Figure 3A) whereas, the distal part of the segment is slightly widened (Figure 3A–C). Seven types of sensilla are found on this segment, including three types of sensilla trichodea (St1, St2, and St3), two types of sensilla basiconia (Sb1 and Sb2), one type of sensilla campaniformia (Sca1), and one type of multilobular sensilla (Sm). Numerous sensilla trichodea (St1) and multilobular sensilla (Sm) are distributed all over the labium II surface (dorsal, lateral, and ventral surfaces). Sensilla trichodea (St1) are distinguished by the presence of many surface pores (Figure 2B,F). One pair of sensilla trichodea (St2) is arranged on the base of segment II. St2 are very long, straight, and almost perpendicular to the surface of the labium (Table 2, Figure 3D,E). The base of the sensillum has a flexible socket, the surface has a vertical groove, and the tip is narrow (Figure 3D,E). Approximately 16 pairs of sensilla trichodea (St3) cover 1/4 of the area of segment II. St3 are long, slightly curved and almost lay flat on the surface of the labium (Table 2, Figure 3D,G). There are three pairs of sensilla basiconica (Sb1) at the base of the second segment (Figure 3D). Sb1 are at middle length, are straight, with a smooth surface, have a base wall pore, a rounded tip, and a flexible socket (Table 2, Figure 3H). Sensilla basiconica (Sb2) are short, small, with a smooth surface, and they have a rounded tip that sits in a pit (Figure 3I). This type of sensillum is sparsely distributed on the ventral surface of the second segment. Sensilla campaniformia (Sca1) are flat, oval-shaped discs with a terminal pore, sparsely distributed on the ventral surface (Table 2, Figure 3F,J).

**Figure 4:**
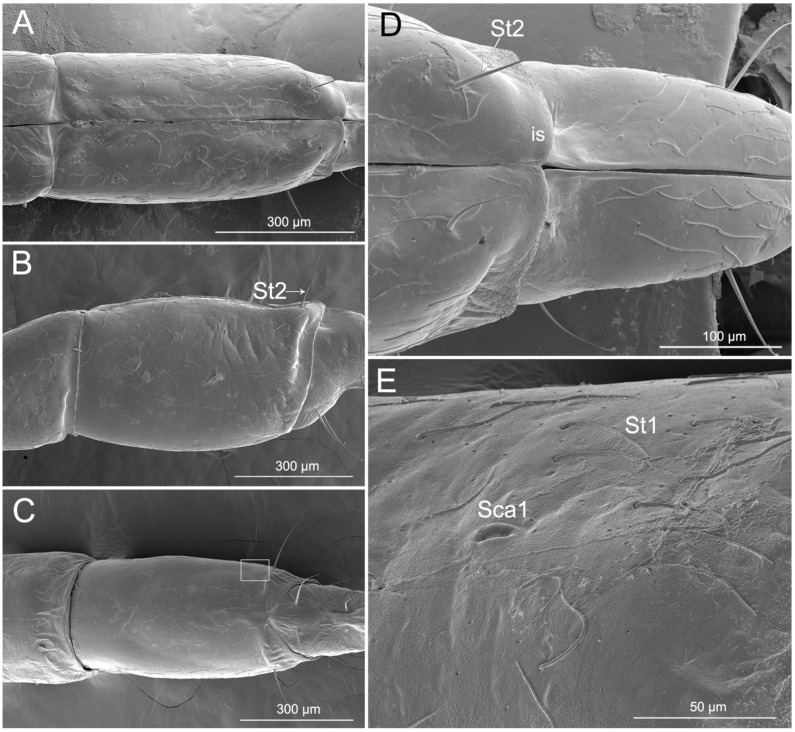
SEM of the third labial segment of *Haematoloecha nigrorufa*. (**A**). Ventral view; (**B**). Lateral view showing sensilla trichodea (St2); (**C**). Dorsal view; (**D**). Enlarged view of dorsal surface showing sensilla trichodea (St2) and intercalary sclerite (is); (**E**). Enlarged view of box in (**C**), showing sensilla campaniformia (Sca1) and sensilla trichodea (St1).

Labial segment III is straight (Figure 4A–C). The latero-dorsal side of the segment is more expanded in the proximal part than in the distal part. The shorter membrane between segments III and IV viewed from the lateral and dorsal side is usually more or less undulate. Four types of sensilla were found on this segment, including two types of sensilla trichodea (St1 and St2), one type of sensilla campaniformia (Sca1), and one type of multilobular sensilla (Sm). Numerous St1 and Sm are distributed all over the segment III surface (dorsal, lateral, and ventral surfaces) (Figure 4A–D). One pair of St2 is found on the distal part of the ventral surface and one pair is found laterally (Figure 4D). One pair of Sca1 is distributed on the dorsal surface (Figure 4E).

**Figure 5:**
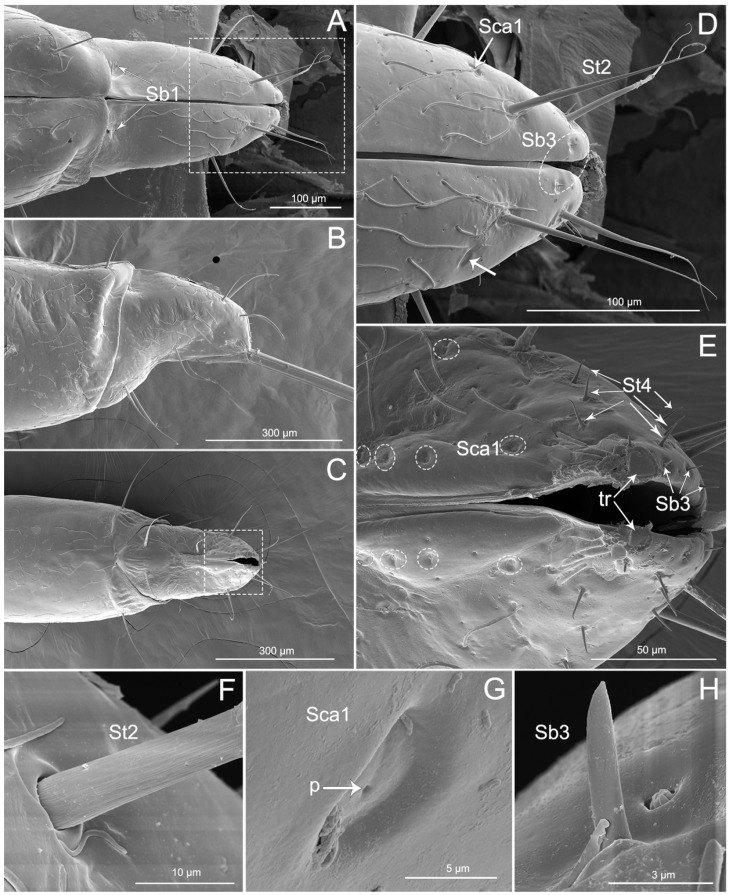
SEM of the fourth labial segment of *Haematoloecha nigrorufa*. (**A**). Ventral view showing a pair of sensilla basiconica (Sb1); (**B**). Lateral view; (**C**). Dorsal view; (**D**). Enlarged view of box in (**A**), showing sensilla trichodea (St2), sensilla campaniformia (Sca1) (white arrows) and sensilla basiconica (Sb3) (white circle); (**E**). Enlarged view of box in (**C**), showing some sensilla campaniformia (Sca1) (white circles), sensilla trichodea (St4) and sensilla basiconica (Sb3); (**F**). Enlarged view of sensillum trichodeum (St2); (**G**). Enlarged view of sensilla campaniformia (Sca1); (**H**). Enlarged view of sensilla basiconica (Sb3) and multilobular sensilla (Sm); *p*, pore.

The labial segment IV is short, blunt, and robust (Figure 5A–C). The shape of the segment is slightly conical, because the ventral side is somewhat convex, in contrast to the lateral–dorsal surface (Figure 5B) with a distinct concavity covering 1/3 of the area. The proximal part of the segment is almost as wide as the end of the third segment (Figure 5B). The end of segment IV is narrowed and slightly bent ventrad, appearing hooklike. A stridulatory organ is composed of two parts, namely a plectrum on the end of the fourth labial segment and a stridulitrum on the prothorax, is present. In the analyzed specimens, the plectrum consisted of one sclerotized tubercle (tr) on each of the paired lateral lobes of the apex of the last labial segment (Figure 5E).

**Figure 6:**
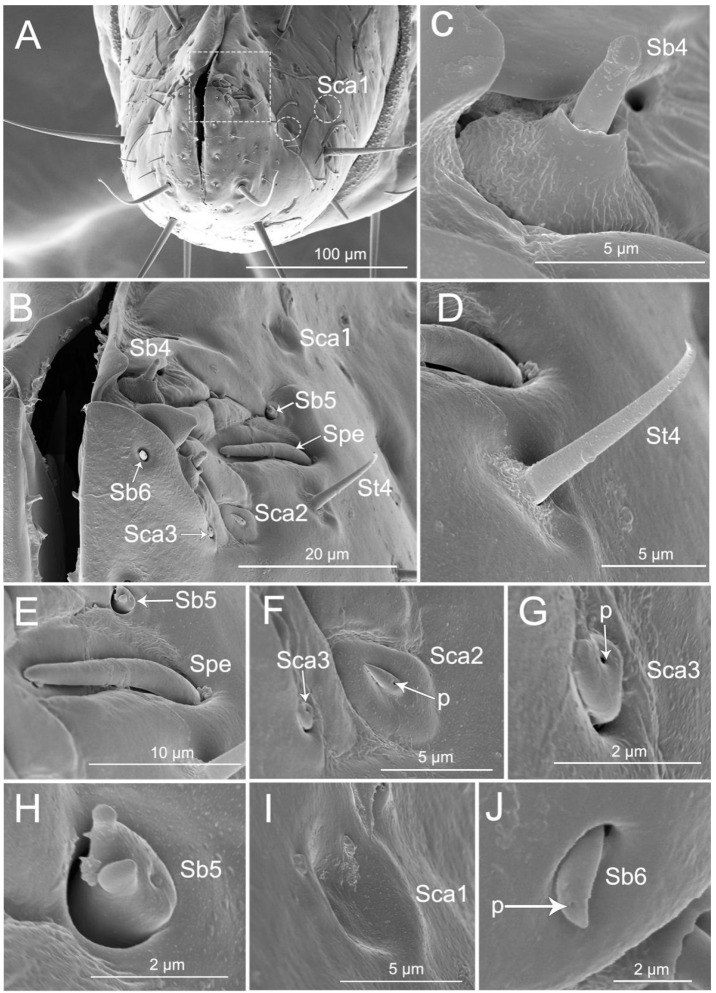
SEM of tip of labium of *Haematoloecha nigrorufa*. (**A**). Vertical view; (**B**). Enlarged view of box in (**A**), showing sensilla trichodea (St4), sensilla placodeum elongated (Spe), three type of sensilla campaniform (Sca1, Sca2, Sca3), and three type of sensilla basiconica (Sb4, Sb5, Sb6); (**C**). Sensilla basiconica (Sb4); (**D**). Sensilla trichodea (St4); (**E**). Placoid elongated sensilla (Spe) and sensilla basiconica (Sb5); (**F**). Sensilla campaniformia (Sca2) and sensilla campaniformia (Sca3); (**G**). Sensilla campaniformia (Sca3); (**H**). Sensilla basiconica (Sb5); (**I**). Sensilla campaniformia (Sca1); (**J**). Sensilla basiconica (Sb6); *p*, pore.

Thirteen types of sensilla were found on this segment, including three types of sensilla trichodea (St1, St2, and St4), five types of sensilla basiconica (Sb1, Sb3, Sb4, Sb5, and Sb6), three types of sensilla campaniformia (Sca1, Sca2, and Sca3), one type of sensilla placoid elongated (Spe), and one type of multilobular sensilla (Sm). There are many sensilla trichodea (St1) and multilobular sensilla (Sm) all over the segment IV dorsal, lateral, and ventral surfaces (Figure 5A–H). Five pairs of sensilla trichodea (St2) are found on segment IV (two pairs on the ventral surface, one pair on the lateral surface, and two pairs on the dorsal surface). Six pairs of sensilla trichodea (St4) are distributed on the proximal part of the fourth segment (Figure 5E). One pair of sensilla basiconica (Sb1) is distributed between the third and fourth labial segments (Figure 5A). Four pairs of sensilla basiconica (Sb3) are found on the distal part of the ventral surface (Figure 5D,E). Eight pairs of sensilla campaniformia (Sca1) are distributed on the proximal position of the last segment (one pair on the ventral surface, two pairs on the lateral surface, and five pairs on the dorsal surface) (Figure 5D,E,G, and Figure 6A).

The labial tip has two lateral lobes and the sensilla are symmetrically arranged on this area and form two sensory fields, including sensilla trichodea (St4), sensilla basiconica (Sb4, Sb5, and Sb6), sensilla campaniformia (Sca1, Sca2, and Sca3), and placoid elongated sensilla (Spe) (Figure 6A–J). Hair-like sensilla trichodea (St4) are short, small, with a smooth surface and they have a rounded tip and a flexible socket (Figure 6B,D). Peg-like sensilla basiconia (Sb3) are short, small, with a smooth surface and they have a narrowed tip and a flexible socket (Figure 5H). Sensilla basiconica (Sb4), similar to sensilla coeloconica, are short cones that arise from inflexible sockets. The base of the sensillum with the socket is elevated above the surrounding cuticle. A sensillum may either extend beyond the socket or remain hidden inside the base (Figure 6C). Sensilla basiconica (Sb5) are a short, flattened cone with additional small processes at the end; the cone base is embedded in a flexible socket (Figure 6H). Sensilla basiconica (Sb6) are short cones with a single pore at the tip and they have a flexible socket (Figure 6B,J). Sensilla campaniformia (Sca2) are flat, oval disks with a single pore on their surface (Figure 6F). Placoid elongated sensilla (Spe) are elongated oval plates that have no pores (Figure 6E).

### 3.4. Stylet Fascicle

The stylet fascicle is composed of two separated mandibular stylets (Md) and two interlocked maxillary stylets (Mx). The mandibular stylets (Md) are slightly shorter than the maxillary stylets (Mx), and right maxillary stylets (RMx) are slightly longer than left maxillary stylets (LMx).

**Figure 7:**
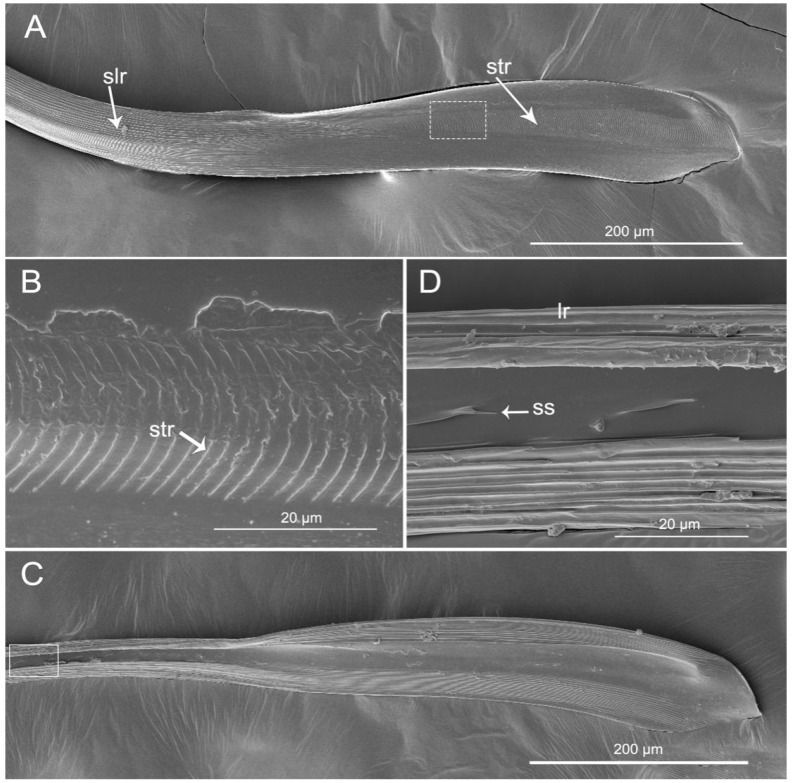
SEM of mandibular stylets of *Haematoloecha nigrorufa*. (**A**). External view; (**B**). Enlarged view of box in (**A**); (**C**). Interior view; (**D**). Enlarged view of box in (**C**); slr, strong longitudinal ridges; str, slightly transverse ridges; lr, longitudinal ridges; ss, small spinule.

The mandibular stylets (Md) are addressed laterally to the maxillary stylets (Mx). In *H. nigrorufa*, the external side of the Md has a spatulate apex with about 150 slightly transverse ridges (str) (Figure 7A,B) and the end is narrowed. Based on the transverse ridges, there are numerous strong longitudinal ridges (slr) that are extended all the way to the base. On the inner surface of the spatulate apex, dorsally, and ventrally are many visible longitudinal ridges (lr). Between them the surface is smooth. Based of the spatulate apex, there are small spikes (ss) on the middle of the inner surface (Figure 7C,D). In this specie, the left-right asymmetry of the maxillary stylets is noticeable.

**Figure 8:**
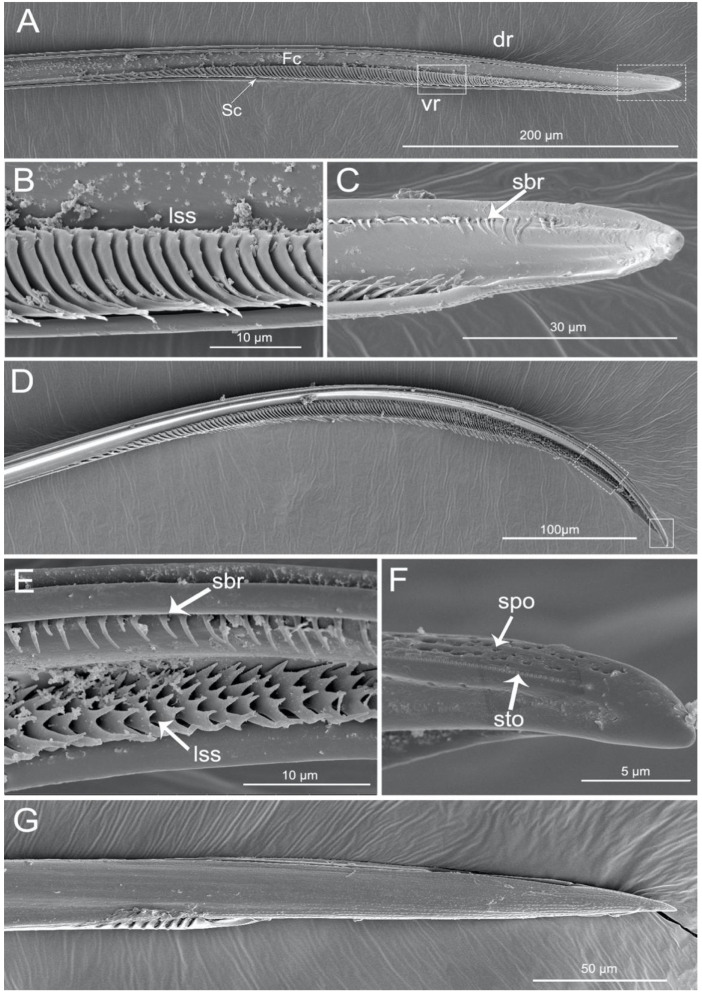
SEM of right maxillary stylets of *Haematoloecha nigrorufa*. (**A**). Interior view; (**B**). Enlarged view of box in (**A**); (**C**). Enlarged view of box in (**A**), showing tip of right maxillary stylet (RMx); (**D**), Lateral view; (**E**). Enlarged view of box in (**D**); (**F**). Enlarged view of box in (**D**), showing tip of right maxillary stylet (Rmx); (**G**). External view; dr, dorsal row; vr, ventral row; lss, lamellate-shaped structures; sbr, short bristles; spo, small pore; sto, small tooth.

**Figure 9:**
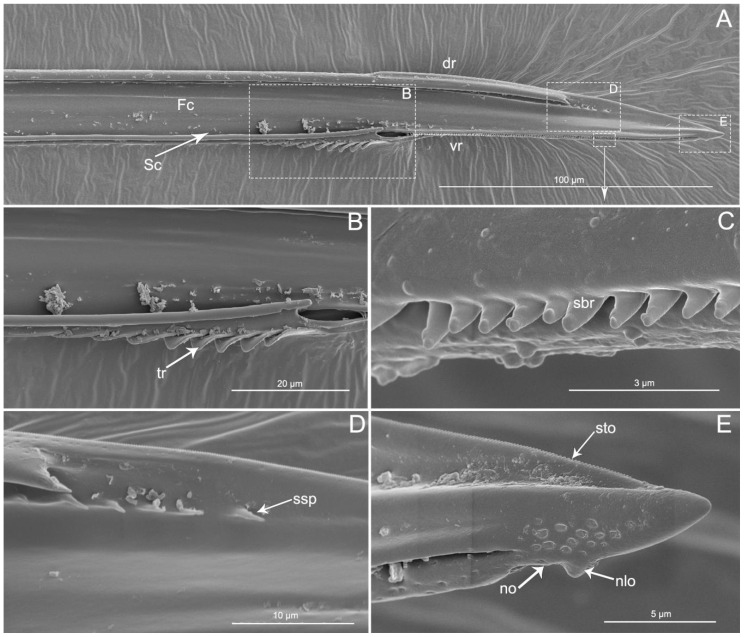
SEM of left maxillary stylets of *Haematoloecha nigrorufa*. (**A**). Interior view; (**B**–**E**). Enlarged view of box in (**A**); tr, transverse ridges; sbr, short bristles; no, notch; nlo, narrow lobe; sto, small tooth; ssp, short spines.

**Figure 10:**
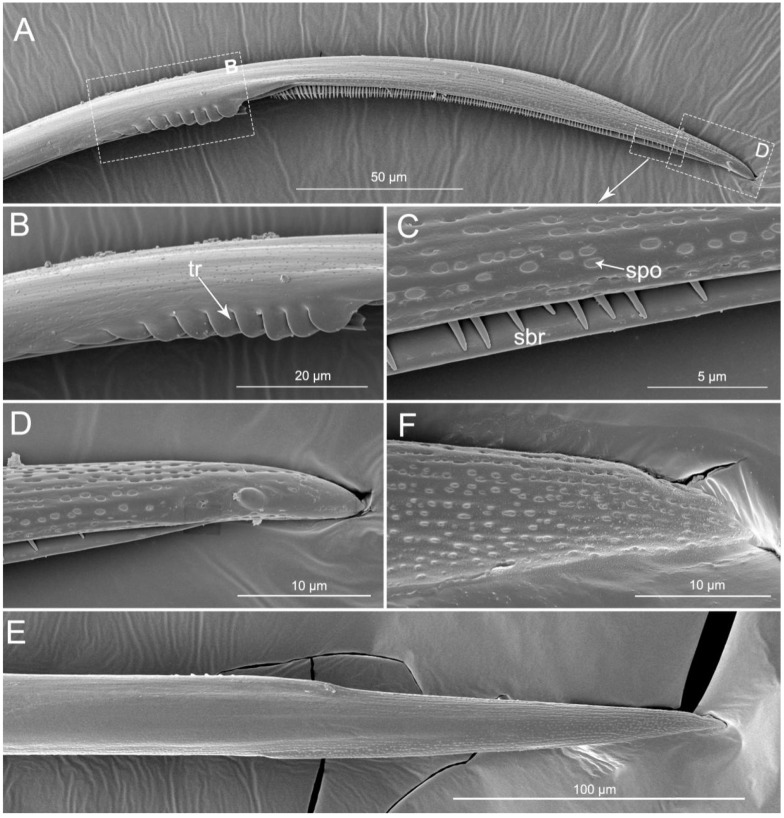
SEM of left maxillary stylets of *Haematoloecha nigrorufa*. (**A**). Lateral view; (**B**). Enlarged view of box in (**A**); (**C**). Enlarged view of box in (**A**); (**D**). Enlarged view of box in (**A**); (**E**). External view; F. Enlarged view of tip of left maxillary stylet; tr, transverse ridges; sbr, short bristles; spo, small pore.

The apex of the right maxilla (RMx) is tapered (Figure 8A,C) and it has a distinct curvature (Figure 8D). The external side of the right maxillary stylet apex of the subapical region is smooth (Figure 8G) with ventral (vr) and dorsal rows (dr) of curved hair-like short bristles (sbr), and the ventral row possesses lamellate structures (lss) (Figure 8A–E). On the lateral surface of the right maxillary stylet (RMx) apex there is a submedial row of many small teeth (sto) (Figure 8F) and a lateral band of many small pores (spo) (Figure 8F). The apex of the left maxilla is straight with a notch (no) and a narrow lobe (nlo) directed anteriorly (Figure 9A,E). On the internal surface of the dorsal (dr) side, there are several short spines (ssp) and many small teeth (sto) (Figure 9D,E). On the ventral (vr) side of the left maxilla, there are some transverse ridges (tr) and short bristles (sbr) (Figure 9A,B, and Figure 10A–C). On the external surface of the left maxillary stylet there are many small pores (spo) (Figure 10C–E).

**Figure 11:**
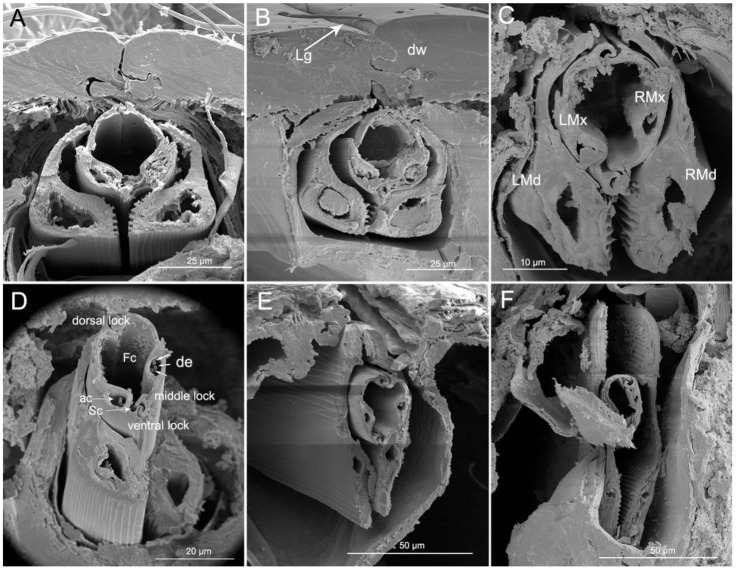
Cross-sections of stylet fascicle and labium of *Haematoloecha nigrorufa*. (**A**). Proximal part of labial segment; (**B**). Median part of segment 2; (**C**). Junction of segments 2 and 3; (**D**). Junction of segments 2 and 3 showing dendrites; (**E**). Median part of segment 3; (**F**). Junction of segments 3 and 4; LMd, left mandibular stylet; LMx, left maxillary stylet; RMd, right mandibular stylet; RMx, right maxillary stylet; Fc, food canal; Sc, salivary canal; de, dendrite; ac, axial canal; Lg, labial groove; dw, dorsal wall.

Cross-sections through the labium and stylet bundle (interlocked maxillae and mandibles) in reduviid species show that the stylet bundles are distinctly laterally compressed (Figure 11A–F). In the cross-section of the labium, the dorsal walls are visible (dw) (edges). The edges are close and they form a tight connection. The floor of the labial groove (lg) is deeper, located under the dorsal edges and envelopes the stylet bundle (Figure 11A,B).

The maxillary stylets are encased by the outer, overlapping mandibular stylets. The shape of the cross-section of the maxillary and mandibular stylets changes from base to end (Figure 11A–F). The two maxillae are held together by interlocking processes forming three locks: dorsal, medial, and ventral. The dorsal lock has two hooked processes and two straight processes. The middle lock has two hooked processes. The ventral lock has one straight and two hooked processes. Within each maxillary stylet there is one axial canal (ac) with three dendrites (de) (Figure 11D). The mandibles are expanded on the ventral side and they possess a wide nerve canal. On the inner ventral wall there is a longitudinal ridge that corresponds to the longitudinal ridges (lr) on Figure 7D. Lateral and dorsal parts of the mandibles are thinner than ventral portions (Figure 11A–D).

### 3.5. The Process of Feeding by Haematoloecha Nigrorufa

*H. nigrorufa* only feeds on millipedes, according to our observations. Both nymphs and adults of this reduviid exhibit negative phototaxis and are gregarious. The adult feeding process involves several steps, including searching and capturing prey, paralyzing prey, a resting phase, and a feeding phase.

Before insects feed, there is a process of finding and capturing prey. When a hungry predator is looking for prey, if the prey is wandering, the time to identify prey is shorter than when the prey is still, since the movement of the prey attracts the visual and the sensory attention of the predator. When this predator senses the presence of millipedes, it will sway the antennae and turn the body to locate the prey. Once the prey has been located, the predator tracks and slowly moves toward the millipede’s head, raises the body, and then captures it as quickly as possible, securing it with the forelegs with stylets oriented toward the substrate. In most cases, the predator captures the prey from behind or from the side.

**Figure 12:**
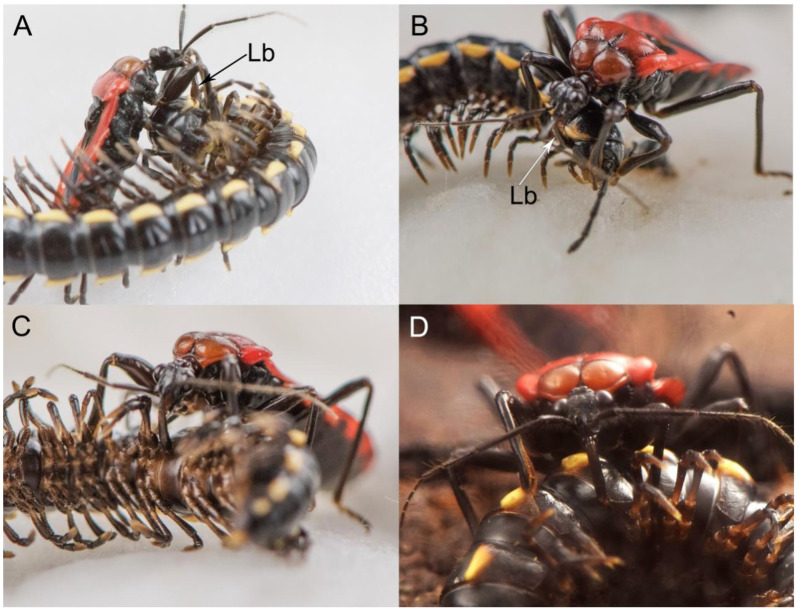
Feeding behavior of adult *H. nigrorufa* on millipede. (**A**). Selecting an appropriate paralyzing site; (**B**). Paralyzing millipede; (**C**,**D**). Feeding on millipede; Lb, labium.

When the prey is caught, the predator uses the forelegs and midlegs to immobilize the prey and pierces the head-collum intersegmental membrane of the prey with its proboscis. In this process, the predator keeps its proboscis perpendicular to the site of paralysis (Figure 12A). It then presses the tip of the labium onto the paralyzing site and then inserts the stylets (Figure 12B). During this process of anesthetizing, the prey’s body contorts acutely, while the predator clings to the millipede’s head for three to 10 minutes. Following paralysis, a resting period of five to fifteen minutes occurs. During this period, the predator might crawl around, clean the antennae and rostrum with the forelegs, and drag the immobilized prey. This cleaning of the body is essential in view of the fact that millipedes, which are preys of these insects, discharge a variety of defensive repellant secretions.

Before the feeding begins, the predator drags the prey to a concealed place. The feeding site of these insects is usually at the intersegmental membrane in the ventral or ventrolateral area. When the predator begins to feed, the beak swings out and the angle between the head and the beak is obtuse (Figure 12C,D). They press the tip of the labium onto the feeding site, insert the stylet, and stay still for about five minutes, and then rest for a while before moving to another part of the body to feed again. The whole feeding process might last from one hour to more than four hours. The labium does not bend and the stylets do not detach from the labium during the entire feeding process. After feeding, the rostrum returns to its resting position along the sternum.

If the predator is disturbed while feeding, it will drag its prey to a safer place and continue feeding. After feeding, it leaves the prey’s body behind in order to rest and clean their antennae in a dark hiding place. Most millipede prey are left with just the exoskeleton, with all soft internal tissues having been consumed by the reduviid.

## 4. Discussion

The overall morphological features of the mouthparts of *H. nigrorufa* are similar to those of other reduviid predators [51,56], including the shape of the labrum, the number of segments of the labium, and the stylet fascicle being composed of two separated mandibular stylets (Md) and two interlocked maxillary stylets (Mx). According to Hwang and Weirauch [57], factors other than microhabitat association may have driven the diversification of Reduviidae; among these are prey specialization and changes in prey capture behavior. In this study of the mouthpart structures in the Reduviidae (Ectrichodiinae) species *H. nigrorufa*, we focused on detailed morphological features that may be related to their unique adaptation for millipede-feeding.

### 4.1. The Mouthparts of A Millipede Specialist

The detailed structure of the labrum is rarely discussed in studies of mouthpart morphology. Spooner [48] reported three different shapes of the labrum (broad and flap-like; long, narrow, and triangular, and broad and flap-like with a long epipharyngeal projection) in Heteroptera. Some previous authors showed that the shape of the labrum might be used for characterization of different Heteroptera taxa [46,48,49,50,52]. In 1969, Štys [50] described several shapes (spiniform, truncate, extremely narrow, and elongate) of the labrum of some Reduviidae, but another unusual condition of the labrum was found in a species of *Ectrychotes* (Ectrichodiinae): the division by a conspicuous transverse membranous zone into two equally well sclerotized parts, called the basilabrum and distilabrum. This labrum structure has been used to characterize the millipede assassin bugs [42,46,52]. In our study we observed that the labrum of *H. nigrorufa* is divided by a transverse membrane into a wide and plicated basilabrum (bl) and an elongated, cone-shaped distilabrum (dl). The non-sclerotized line dividing basilabrum from distilabrum in *Ectrychotes* [50] and other studied taxa (*Nularda nobilitata, Ectrichodiella minima*) [42] and *H. nigrorufa* may be, either a novel trait or a remnant of an intermediate stage in the evolution of a long labrum by means of sclerotization of the epipharynx [50]. As suggested by Spooner [48] and Štys [50], a broad, flap-like, simple labrum is probably a primitive feature of Heteroptera. Among reduviids, a subdivided labrum is only characteristic of Ectrichodiinae and Triatominae [42], both unique trophic specialists. Weirauch [42] suggested that a subdivided labrum is of independent origin in Ectrichodiinae and Triatominae, and synapomorphic for both groups. Moreover, the labrum of the studied species shows significantly different composition of sensilla when compared to other reduviid species and this aspect is discussed in the following section on types and functions of sensilla.

The labium of *H. nigrorufa* consists of three visible segments; but, the two subfamilies Hammacerinae and Centrocneminae with four-segmented labia in Reduviidae [42,43,58,59,60]. In most Reduviidae the first segment is deemed to be either lost or fused to the head capsule [43], which suggested that the four-segmented labium in Hammacerinae is plesiomorphic and homologous to those of non-reduviid Cimicomorpha Weirauch [43]. In most taxa of Heteroptera, the labium is four-segmented, and this feature is used in the classification of the true bug taxa [43,48,60]. The labium of *H. nigrorufa* generally is similar to those of other reduviid species in the number of segments and membrane connections between them. However, another feature in this species is the shape of the last segment. Our SEM observations showed that the segment is short, bent dorsad, and hooklike, differing from other species of Reduviidae, in which the last segment is straight or slightly curved, long or short [61].

We consider the hook-shaped last segment of *H. nigrorufa* to be a special adaptation to feeding on millipedes, although the labium of heteropterans plays an indirect role in predation (maintaining the bundle of stylets, to act as a guide as the stylets are pushed into host/prey tissue) [10,23]. Members of Ectrichodiinae usually approach the millipede’s head and paralyze their prey by inserting the stylet at the head-collum intersegmental membrane, according to descriptions of feeding behavior [62,63,64,65,66]. Moreover, if the assassin bug is disturbed, it will drag the prey to a safer place and continue feeding. In both situations, the hook-shaped segment of *H. nigrorufa* seems to be helpful because its curved shape facilitates the perpendicular insertion of the stylets to the membrane between the head and collum, as well as assists with dragging the victim’s body. During an attack, the assassin bug clings to the millipede’s head (for about four minutes) and the head-collum region is frequently selected to avoid inserting stylets into laterally or dorsally located defensive glands along the trunk during millipede immobilization and consumption [53].

The stylets are the main feeding organs and show great differences among groups with different feeding habits in Heteroptera [12,15,16,19,23,24,35,36,42,45,46,51,56,67,68,69,70,71,72,73,74]. The subfamily of Ectrichodiinae has a strong preference for feeding on millipedes [53,75], but detailed research on the mouthpart structures in this group were previously lacking. We only found general data of morphology of the stylets in previous papers by Cobben [12], Weirauch [42], and Forthman and Weirauch [46] described for some species based on light or SEM microscope studies. The present study reveals several new characters of the mandibular stylets in *H. nigrorufa*, e.g., a greatly expanded apex with abruptly tapered tip and 150 approximately transverse ridges on the external middle side. Moreover, on the inner side of the right and left mandibles are a row of cuticular spikes and longitudinal ridges (lr). These longitudinal ridges are used to lock the two mandibular stylets together below the ventral side of the maxillae; they are more visible in cross section (Figure 11). The maxillary stylets are enclosed in the spaces between the inner longitudinal ridges. A similar spatulate shape of the mandibles is present in other Ectrichodiinae, *Brontosioma discus* Burm [12], and *Nularda nobilitata* [42], but the transverse ridges are usually less numerous (up to 35 or more than 35) and they apparently lack spikes on the inner surface. Similar spatulate mandibles, but lacking the transverse ridges on the outer surface, were observed in some species of Harpactorinae and Sphaeridopinae [12]. The greatest similarity in mandible shape occurs between Ectrichodiinae and Tribelocephalinae [42]; the latter group feeds on ants, termites, and blattids [75]. Both taxa are characterized by rather faint transverse ridges on the outer stylet surface. Based on combined phylogenetic results, Tribelocephalinae were synonymized with Ectrichodiinae [46]. Presumably, transverse ridges help to keep the stylets firmly anchored in prey tissues during the initial attack when the prey may be struggling. Perhaps the larger number of ridges in the studied species is related to the tendency of millipedes to struggle more vigorously after being attacked than the prey of other reduviids with fewer or no ridges.

The left-right asymmetry of the maxillary stylets in *H. nigrorufa* is noticeably similar to that found in most heteropterans, especially predators [12,19]. Previous studies indicate that the right maxillary stylets of predacious bugs usually possess more barb rows [12,15,16,19,42,45,46,51,56,68,69]. Our study is consistent with these previous observations. The characteristic of the right maxilla appear as lamellate structures in the internal ventral side. The lamella are only present in other species of Ectrichodiinae and Tribelocephalinae [42]. Many of the different curved hair-like processes observed in *H. nigrorufa* are also usually present in other true bugs [19,46,68]. Because the hair-like and bristle-like structures are numerous and localized in ventral and dorsal rows (Figure 8A–E), their distribution is similar to those of other heteropterans, except those taxa with reduced hairs, such as Triatominae [42]. The left stylet in the studied species has fewer internal hair-like processes. Frequently the hair system of both maxillae is called a grating-system [12]. Because the food in the suction stylets is semi-fluid substances and particulate matter, which result from the lacerating effects of the spines extending from the maxillae, it must be filtered through the underlying grating structures. In *H. nigrorufa*, bigger spikes and very small edge files are present on the outer side of the left stylet. This mentioned set of hair-like structures is only similar to other Ectrichodiinae. Thus, as suggested by Weirauch [42] and Forthman and Weirauch [46], more extensive comparative studies of the morphology of the maxillary stylets may provide useful taxonomic characters.

Cross sections of the stylets show that the fine-structure and interlocking mechanism of the maxillae of *H. nigrorufa* are similar to those of other reduviid taxa [12,47].

### 4.2. Labial Sensillar System

Detailed morphological descriptions of Ectrichodiinae (Reduviidae) labial sensilla have never been previously reported. The labium of hemipterans plays an important role in not only receiving the stylet fascicles, but also in the detection of the host by the sensory structures present on the surface [6,10]. The various sensilla, distinguishable by their different external morphological characters perform different functions including chemosensory (gustatory and olfactory), thermo-hygroreceptive, proprioceptive and mechanosensory. In this study, fifteen types of sensilla were observed on the mouthparts of the reduviid predator *H. nigrorufa*.

The most abundant sensilla on the labium in this species are three kinds of sensilla trichodea, which have no pores, a more or less flexible shank, and the base embedded in a flexible socket (St2, St3, and St4), and are considered to be mechanoreceptive. Mechanoreceptors also include sensilla basiconica type Sb1 with a proprioceptive function and sensilla campaniformia (Sca1, Sca 2, and Sca 3). New in this study is the report of sensilla trichodea (St1) with porous walls, which occur in large numbers on the labium and labrum. These sensilla are considered to be chemoreceptive (olfactory). In other species of Ectrichodiina,e the sensilla on the labium were not studied, so it is not yet known whether these sensilla represent a special adaptation that is related to feeding on millipedes. This type of sensilla was also not observed in studies of the labial sensilla in Triatominae and Peiratinae [18,44,76]. The sensitivity and chemical range of insect olfactory systems is remarkable, enabling them to detect and discriminate a wide range of different odor molecules. There is a striking evolutionary convergence towards a conserved organization of signaling pathways in all insect olfactory systems, because the olfactory transduction and neural processing in the peripheral olfactory pathway involve basic mechanisms that are universal across species [77]. Such functioning of the olfactory system does not exclude olfactory sensillae of a different shape than those listed e.g., St1 may also be included in this system. We can only assume that in the studied species (or all species of Ectrichodiinae) only one type of sensilla (St1) on the labium responds to odors of their millipede prey, as suggested by their presence and abundance in these millipede specialists, but absent in related groups of Reduviidae. Unfortunately, the antennal system sensilla in this group of reduviids has not been studied, so data of other types of olfactory sensillae are unknown.

On the labial tip in most heteropterans, there is usually a singule olfactory sensillum basiconicum and/or sensillum placodeum. These sensilla are arranged in a pattern that is highly stereotypical among most heteropterans [15,16,18,19,21,23,24,25,26,27,28,29,30,31,32,72,78].

The thermo-hygro receptive sensilla identified as sensilla basiconica (Sb2) and multilobular sensilla (Sm). The flavor sensilla located near the plectrum, which includes three types—Sb4, Sb5, and Sb6—and placoid elongated sensilla (Spe). The described shapes and functions of sensilla, except for sensilla trichoidea (St1) in *H. nigrorufa*, conform to the general model of the labial sensilla present in other reduviids [18,44,76]. However, slight differences may be observed among taxa, especially in the quantity and size of sensilla. The present morphological and functional classifications of the labial sensilla in *H. nigrorufa* are in accordance with the features described for the mentioned types and functions of sensilla reported by many authors [18,24,25,44,54,55,79].

## 5. Conclusions

This study provides the first detailed fine-structural characterization of the unique mouthparts in *Haematoloecha nigrorufa* (Ectrichodiinae), including the location and distribution of different sensilla types. Judging from the morphology and function, the basal set of types/subtypes of the labial sensilla of *H. nigrorufa* does not strongly differ from other species of reduviids, meaning that a similar pattern of sensilla is visible. However, in particular, we report the presence of more numerous olfactory sensilla trichodea (St1) on the labium and labrum in comparison to the other types of olfactory sensilla in reduviids and other heteropteran taxa. There is also novelty in the special shapes of the labrum, the hook-shaped ultimate segment of the labium, and the large spatulate apex with the many transversal shallow grooves on the external side of the mandibles. These mentioned structures may represent specialized adaptations that are related to the millipede feeding.

## Figures and Tables

**Table 1:** Measurements of labrum, labium and stylets (mean ± SE) obtained from scanning electron microscopy. Lb, labium; Lb, 2, 3, 4, the second, third, fourth segment of labium; Lm, labrum; Md, mandibular stylets; RMx, right maxillary stylet; LMx, left maxillary stylet; *N* = sample number.

Sex	Position	Length (μm)	Width (μm)	*N*
Female	Lb	2058.0 ± 25.8	-	12
	L2	1174.8 ± 35.9	215.3 ± 4.4	12
	L3	587.4 ± 15.7	301.7 ± 5.8	12
	L4	298 ± 18.3	194 ± 2.8	12
Male	Lm	476.5 ± 8	-	4
	Lb	1875.2 ± 113.5	-	3
	Lb2	1011 ± 131.2	219 ± 8.7	3
	Lb3	583.6 ± 14.2	288.2 ± 1.2	3
	Lb4	285.8 ± 18.9	183 ± 4.4	3
	Md (spatulate apex)	507.4 ± 15.3	-	4
	RMx (dorsal short bristles)	104.3 ± 6.5	-	3
	RMx (ventral lamellate structures)	424.2 ± 13.7	-	3
	LMx (dorsal short spines)	15.8 ± 0.5	-	3
	LMx (ventral short bristles)	76.4 ± 3.6	-	3

**Table 2:** Distribution, morphometric data (mean ± SE), terminology and definition of sensilla used in the present paper after data of prior authors [18,48,49]. Lb2, 3, 4, the second, third, fourth segment of labium; Lm, labrum; *N* = sample size; St1–4, sensilla trichodea I-IV; Sb1–6, sensilla basiconica I-VII; Sca1–3, sensilla campaniformia; Spe,placoid elongated sensilla; Sm, multilobular sensilla; SF, sensory field on the labial tip; Wp, wall pore; Tp, tip pore.

Type	Distribution and Number	Length (μm)	Basal Diameter (μm)	Shape	Socket	Surface	Pore	Category	Function	*N*
St1	Lm, Lb2-Lb4	42.8 ± 1.7	1.45 ± 0.1	Hair in pit	Fnflexible	Smooth	Wp (Multiporous)	Chemoreceptive sensilla	Olfactory	20
St2	Lb2 (1 pair), Lb3 (2pairs), Lb4 (5pairs)	142.8 ± 6	5.3 ± 0.2	Hair, peg	Flexible	Grooved	No	Mechanoreceptive sensilla	Tactile	11
St3	Lb2 (about 16 pairs)	103.8 ± 5.8	5.2 ± 0.1	Hair	Flexible	Grooved	No	Mechanoreceptive sensilla	Tactile	10
St4	Lb3 (6 pairs)	9.4 ± 0.6	1.6 ± 0.1	Hair, peg	Flexible	Smooth	No	Mechanoreceptive sensilla	Tactile	14
Sb1	3 pairs at the base of the second segment, 1 pair on the junction between the third and fourth segment	32.7 ± 2.5	4.9 ± 0.1	Peg	Flexible	Smooth	Wp (Uniporous)	Proprioceptive sensilla	Perceive the degree of flexion of the joint	10
Sb2	Lb2 (2 pairs)	9.5 ± 0.7	1.4 ± 0.1	Peg in pit	Inflexible	Smooth	No	Thermo-hygroreceptive sensilla	Temperature/humidity	6
Sb3	Lb4 (4 pairs)	6.0 ± 0.4	1.1 ± 0.1	Peg	Flexible	Smooth	No	Mechanoreceptive sensilla	Tactile	14
Sb4	SF (2 pairs)	5.1 ± 0.4	4.2 ± 0.2	Peg (sensillum coleoconicum)	Inflexible	Smooth	Tp	Chemoreceptive sensilla	Gustatory	8
Sb5	SF (1 pair)	1.3 ± 0.1	1.2 ± 0.1	Peg	Flexible	Smooth	Tp	Chemoreceptive sensilla	Gustatory	4
Sb6	Tip (1 pair)	1.7 ± 0.1	1.2 ± 0.1	Peg in pit	Flexible	Smooth	Tp	Chemoreceptive sensilla	Gustatory	4
Sca1	Lb2-Lb4	-	7.2 ± 0.7	Oval plate	Inflexible	Smooth	Tp	Proprioceptive sensilla	Perceive the degree of flexion of the joint	8
Sca2	SF (1 pair)	-	5.5 ± 0.3	Dome-like structures	Inflexible	Smooth	Tp	Proprioceptive sensilla	Perceive the degree of flexion of the joint	4
Sca3	SF (1 pair)	-	1.2 ± 0.1	Oval plate	Inflexible	Smooth	Tp	Proprioceptive sensilla	Perceive the degree of flexion of the joint	4
Spe	SF (1 pair)	16.8 ± 2.1	2.2 ± 0.1	Dome-elongated	Inflexible	Smooth	Tp	Chemoreceptive sensilla	Gustatory	8
Sm	Lm, Lb2-Lb4	-	1.5 ± 0.1	Pegs in cavity surrounded by fingerlike structures	Inflexible	Smooth	No	Thermo-hygroreceptive sensilla	Temperature/humidity	20
